# Tigecycline Tango: A Case of Antibiotic-Induced Pancreatitis

**DOI:** 10.7759/cureus.44538

**Published:** 2023-09-01

**Authors:** Babu Sriram Maringanti, Venu M Ganipisetti, Scott S Jun, Mario I Flores

**Affiliations:** 1 Hospital Medicine, University of New Mexico, Albuquerque, USA; 2 Hospital Medicine, Presbyterian Hospital, Albuquerque, USA; 3 Internal Medicine, University of New Mexico, Albuquerque, USA

**Keywords:** antibiotic associated pancreatitis, tigecycline induced acute pancreatitis, drug induced pancreatitis, acute pancreatitis, tigecycline

## Abstract

Acute pancreatitis is a frequent cause of hospitalization, with the most common triggers being alcohol consumption and gallstones. Although the incidence of drug-induced pancreatitis remains low, it is steadily increasing due to the advent of newly discovered broad-spectrum antibiotics targeting multi-drug resistant organisms. Tigecycline, a broad-spectrum intravenous antibiotic derived from the tetracycline class, was approved by the FDA in 2005 for the treatment of complicated skin and skin structure infections, complicated intra-abdominal infections, and community-acquired pneumonia. It has activity against vancomycin-resistant Enterococcus, Methicillin-resistant *Staphylococcus aureus*, multi-drug-resistant *Acinetobacter baumannii*, multi-drug-resistant *Stenotrophomonas maltophilia*, and Extended Spectrum Beta-lactamase (ESBL) producing Enterobacter species. However, it was later discovered that tigecycline can cause acute pancreatitis. We present a case of a 27-year-old female patient who was admitted to the emergency department with abdominal pain and was subsequently diagnosed with tigecycline-induced pancreatitis based on the clinical resolution after withdrawal of the drug.

## Introduction

Acute pancreatitis is the leading gastrointestinal cause of hospitalization in the US [1]. Gallstones and alcohol are the most common causes of acute pancreatitis [1]. Although drug-induced pancreatitis remains rare (accounting for less than 3% of cases), it has been increasingly recognized [2]. Understanding the underlying cause of acute pancreatitis is essential, as it guides both treatment and prevention efforts. These etiologies can often be identified through patient history and initial laboratory results in the emergency department. However, when these common causes have been ruled out, a thorough review of the patient's history and medication list may be necessary to uncover an unusual cause such as medication-induced pancreatitis. Although the incidence of tigecycline-induced pancreatitis remains very low (less than 1%) [3], it is imperative to have a suspicion and subsequent plan to discontinue usage of this drug as soon as possible when no obvious common etiologies are discovered. In our patient’s case, after ruling out other likely causes, tigecycline emerged as the most probable reason for her pancreatitis.

## Case presentation

A young female in her late 20s with a past medical history of hypothyroidism, asthma presented to the emergency department with a chief complaint of acute abdominal pain.

Approximately one year prior to presentation, the patient underwent a Roux-en-Y bypass for obesity, and her post-surgical course was complicated by recurrent skin and soft tissue infections at the laparoscopic site requiring surgical drainage at an outside hospital. Cultures from drainage returned positive for *Mycobacterium abscessus*. The patient was being treated by an infectious disease specialist as an outpatient. She was initially placed on doxycycline and levofloxacin which were discontinued due to intolerance and switched to amikacin and tigecycline pending sensitivities. The patient has been on tigecycline for about 2 weeks prior to presenting symptoms.

During our interview with the patient, she reported having a 3-4 day history of progressively worsening epigastric and left lower quadrant abdominal pain. Her pain was described as sharp with radiation to her back with no alleviating factors. She also reported having associated nausea, vomiting, and decreased tolerance for oral intake over this time.

The patient has a past surgical history of cholecystectomy and denied having any alcohol, tobacco, and drug use. Home medications included amikacin, tigecycline, levothyroxine, montelukast, PRN albuterol, calcitriol, and calcium carbonate. She reported drug allergies to non-steroidal anti-inflammatory drugs (NSAIDs) and oxycodone and food allergies to bananas and peanuts.

**Figure 1:**
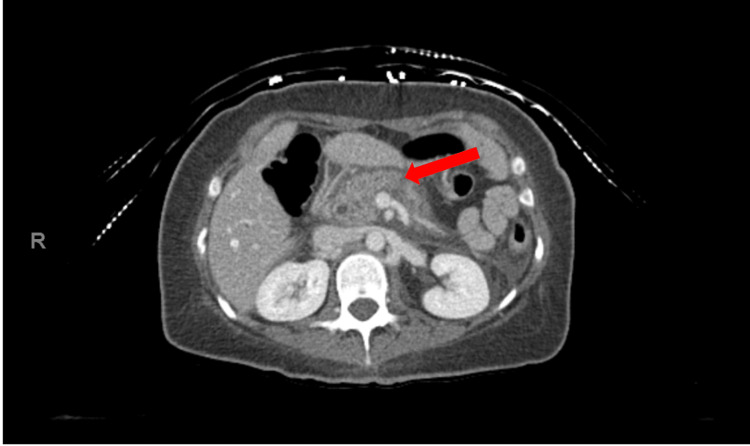
Abdominal and pelvic computed tomography (CT) showing interstitial pancreatitis (red arrow).

The patient’s vitals were notable for a heart rate of 113 bpm but otherwise were stable. Tenderness in the epigastric region was noted with deep palpation, otherwise, the physical exam was unremarkable. Labs were notable for WBC 28.5 x 103/uL, Lipase 1,621 Unit/L. The patient’s lipid panel and liver function tests including aspartate transaminase (AST), alanine aminotransferase (ALT), alkaline phosphatase, and total bilirubin were all within normal limits. CT scan of the patient’s abdomen revealed interstitial pancreatitis (refer to Figure 1 showing CT image with a red arrow pointing towards pancreatitis) which was complicated by a non-occlusive thrombus of the splenic vein. It also showed findings of prior cholecystectomy with mild biliary prominence favored post-cholecystectomy effect. Common bile duct measured 6 mm. The patient was empirically started on ceftriaxone and metronidazole for possible abdominal infection. Once the leukocytosis was thought to be reactive and there was no evidence of necrotizing pancreatitis, the antibiotics were discontinued. Therapeutic enoxaparin was started along with aggressive intravenous fluid resuscitation, and an infectious disease consult was placed.

Upon admission to the medical ward, the inpatient infectious disease (ID) team communicated with the patient’s outpatient infectious disease specialist and confirmed that she was receiving treatment with tigecycline and amikacin for a draining abdominal wall wound that had grown *M. abscessus*. 

Given that there was no other clear etiology for the patient's pancreatitis (no alcohol use, prior cholecystectomy, and no laboratory and imaging evidence of biliary stones, no hypercalcemia, no hypertriglyceridemia, no signs of viral illness), the most likely culprit was the tigecycline which was started about 2 weeks prior to presentation. The patient rapidly improved over the 24 hours following admission to the hospital with supportive care. The decision was made to permanently discontinue tigecycline and start imipenem. Amikacin was continued as it is not known to cause pancreatitis. The patient was discharged after 2 days with a close follow-up with her outpatient ID specialist. 

Upon review of the medical record, the patient did not have a recurrence of acute pancreatitis thus far (10 months since the first episode at the time of writing this article).

## Discussion

Drug-induced pancreatitis (DIP) is estimated to occur in 1-2% of all episodes of acute pancreatitis [2]. Tigecycline, a derivative of the tetracycline class of drugs, was first approved by the Food and Drug Administration in 2005 for the treatment of various types of infections, such as skin and soft tissue infections, intra-abdominal infections, and bacterial pneumonia. Developed to overcome the common mechanisms of resistance such as ribosomal protection and efflux pumps [3], tigecycline's safety profile initially listed adverse effects such as nausea, vomiting, and diarrhea. However, it wasn't until a retrospective cohort study and a review of phase III trials that pancreatitis was identified as a potential adverse effect [4]. With the rise of multidrug-resistant organisms, tigecycline's ability to inhibit these organisms in vitro has led to its off-label use for the treatment of these infections [4] and may be contributing to the rise in the incidence of pancreatitis. Several mechanisms have been proposed to explain the link between tigecycline use and pancreatitis, including the production of toxic metabolites, elevation of triglyceride levels, and high drug accumulation in the biliary system [3]. 

Nison Badalov et al [5] classified drug-induced pancreatitis into four classes of drugs based on the level of evidence. According to this, tigecycline can be categorized under class I drug where there is at least 1 case report describing the recurrence of pancreatitis after rechallenging with the drug [6].

There are currently no established diagnostic criteria for this condition. To help identify this condition, some experts have recommended using a combination of guidelines such as the onset of pancreatitis during tigecycline therapy, exclusion of other common causes of pancreatitis, and improvement of symptoms after discontinuation of the drug [7]. 

In a literature review of 11 case reports published in Dec 2021 byPeng-fei Wang et al [3], almost all cases of acute pancreatitis developed within about 2 weeks of initiation of tigecycline except one case report where it was after 4 weeks [8]. In our case, the patient started experiencing symptoms of acute pancreatitis on day 12 of tigecycline use, and was admitted to the hospital on day 14 of tigecycline treatment. Tigecycline was discontinued on day 14 and the patient's symptoms improved by day 16.

Naranjo probability scale is a widely used scoring system to assess the causal relationship between an adverse event and the drug in question [9]. The Naranjo score for our patient is 7 which falls under the category of probable association. Rechallenging our patient with tigecycline would have provided more evidence but was not done due to more risks versus benefits in our patient given that there were other antibiotic options to treat her skin and soft tissue infection.

## Conclusions

Acute pancreatitis is the most common gastrointestinal cause of hospitalizations in the United States. As healthcare providers, it is crucial to consider all possible etiologies, including DIP, once the more common causes have been ruled out. Among the drugs associated with DIP, tigecycline, a broad-spectrum antibiotic, is a known but uncommon cause. The underlying mechanism behind tigecycline-induced pancreatitis is not fully understood, and more research is needed to fully comprehend the potential synergistic toxic effects that may be associated with this medication when taken in combination with other drugs. Furthermore, additional studies can reveal which patient population remains at risk and which denominators can affect patient care and outcome. Therefore, it is essential for healthcare providers to remain vigilant for tigecycline-induced pancreatitis and to carefully consider the potential benefits and risks of tigecycline use for each individual patient.
